# Can Lactate Values Predict Postoperative Atrial Fibrillation Following Coronary Artery Bypass Graft Surgery? A Prospective Observational Study

**DOI:** 10.21470/1678-9741-2024-0440

**Published:** 2026-05-06

**Authors:** Zeki Temiztürk, Osman Fehmi Beyazal, Ahmet Can Topçu, Nihan Kayalar, Mehmed Yanartaş

**Affiliations:** 1 Department of Cardiovascular Surgery, İstanbul Başakşehir Çam and Sakura City Hospital, İstanbul, Turkey.; 2 Department of Cardiovascular Surgery, Kartal Koşuyolu High Specialization Training and Research Hospital, İstanbul, Turkey.

**Keywords:** Atrial Fibrillation, Coronary Artery Bypass Graft, Lactate

## Abstract

**Introduction:**

The aim of our study is to investigate the relationship and predictive significance of routinely measured serum lactate values with postoperative atrial fibrillation (POAF) in patients following isolated coronary artery bypass grafting (CABG).

**Method:**

Between 2022 and 2023, 250 patients who underwent isolated CABG were prospectively examined. Patients were divided into two groups, those with POAF (Group 1) and those without POAF (Group 2). Preoperative and postoperative 0^th^, 2^nd^, 4^th^, 8^th^, and 24^th^-hour lactate values were measured.

**Result:**

POAF was observed in 58 (23.2%) patients. There was no statistical difference between the groups in terms of ejection fraction, number of bypass grafts, cross-clamping time, cardiopulmonary bypass time, drainage, and beating operation rates. No statistically significant difference was found between the preoperative values and postoperative lactate values at zero, two, four, eight, and 24 hours between the groups. No statistically significant difference was found between the groups in terms of the difference between postoperative 0^th^, 4^th^, 8^th^, and 24^th^-hour and preoperative serum lactate levels. No statistically significant difference was found between postoperative 4^th^, 8^th^, and 24^th^-hour and postoperative 0^th^-hour serum lactate levels. Mortality, cerebrovascular accident, length of intensive care unit stay, and hospital stay were higher in Group 1.

**Conclusion:**

We found that early postoperative lactate values were higher in patients with POAF than in those without POAF, although this difference did not reach statistical significance. Lactate values alone are insufficient to predict POAF following CABG.

## INTRODUCTION

**Table:** 

Abbreviations, Acronyms & Symbols				
AF	= Atrial fibrillation		HT	= Hypertension
AKI	= Acute kidney injury		ICU	= Intensive care unit
CABG	= Coronary artery bypass grafting		IQR	= Interquartile range
CAS	= Carotid artery stenosis		PAD	= Peripheral arterial disease
COPD	= Chronic obstructive pulmonary disease		POAF	= Postoperative atrial fibrillation
CPB	= Cardiopulmonary bypass		Postop.	= Postoperative
CVA	= Cerebrovascular accident		RBC	= Red blood cells
EF	= Ejection fraction		XCL	= Cross-clamping

Postoperative atrial fibrillation (POAF) is an important complication that increases morbidity and mortality in cardiac surgery. Its incidence in isolated coronary artery bypass grafting (CABG) is reported to be around 20 - 30%, while it is around 35 - 40% in isolated valve surgery^[1]^. Although many risk factors have been identified, such as advanced age, P wave abnormalities on the electrocardiogram, dilated left atrium, emergency surgery, low ejection fraction (EF), low glomerular filtration rate, and chronic obstructive pulmonary disease (COPD), there is no definitive consensus on this issue. POAF has been associated with hospital cerebrovascular accident (CVA), acute kidney injury (AKI), heart failure, prolonged intensive care unit (ICU) and hospital stay, increased costs, and death^[2]^.

It is important to identify patients at high risk of POAF to apply preventive and therapeutic modalities to the right patients. Lactate is the end product of anaerobic glycolysis, and its level in serum increases in cases that cause hypoxia at the tissue level. Serum lactate values, regularly measured in routine cardiac surgery practice, are an indirect indicator of cardiac output^[3]^. In this study, we investigated the relationship and predictive significance of routinely measured serum lactate values with POAF in patients undergoing isolated CABG.

## METHODS

This study was designed as a single-center, prospective, observational case-control study. Patients over the age of 18 years who underwent isolated CABG between January 1, 2022, and January 1, 2023, at the Cardiovascular Surgery Clinic of Istanbul Basaksehir Çam and Sakura City Hospital were included in the study. Patients with a history of preoperative atrial fibrillation (AF), those who underwent additional cardiac procedures other than CABG, those with more than mild valve stenosis or insufficiency, and those undergoing emergency operations were excluded from the study. As a result of the exclusion criteria, a total of 250 patients were included in the study.

The patients' basic demographic data, comorbidities, operative details, amount of bleeding and blood product replacements, preoperative and postoperative serum lactate levels, and post-operative adverse events were recorded. Then, the patients were divided into two groups: Group 1, patients with POAF, and Group 2, patients without POAF. Serum lactate values measured on the day of surgery were accepted as preoperative values. In addition, serum lactate levels were measured at zero, two, four, eight, and 24 hours postoperatively, and the differences between them were examined. The current parameters measured were compared between these groups, and the relationship between serum lactate values and other parameters and POAF was investigated. Lactate values were collected using The BD A-Line™ Arterial Blood Collection Syringes and analyzed immediately using the ABL90 FLEX blood gas analyzer.

This study was approved by the Istanbul Basaksehir Çam and Sakura City Hospital Ethics Committee (decision no: 2021-289).

## Statistics

Data were analyzed by using IBM SPSS Statistics for Windows, version 20.0 (IBM Corp., Armonk, N.Y., USA). Continuous variables in the study were presented as minimum, maximum, median, and interquartile range. Categorical variables were expressed as numbers and percentages. The normality of distribution was assessed by the Kolmogorov–Smirnov test. For numerical variables, differences between patients and controls were tested using *t*-test for parametric data or the Mann–Whitney U test for non-parametric data. Categorical variables were analyzed using the Pearson χ^2^ test and Fisher’s exact test for parametric and nonparametric data, respectively. The level of statistical significance was set at *P* < 0.05.

## RESULTS

The basic demographic data, clinical characteristics, and perioperative data of the patients are shown in Table 1. POAF was observed in 58 (23.2%) of 250 patients. When the groups were compared, no difference was observed in terms of sex, but the median age of Group 1 was higher than that of Group 2 (66 – 59 years, respectively, *P* =0.00). There was no significant difference in terms of diabetes mellitus, COPD, chronic kidney disease, or peripheral artery disease between the groups, but hypertension (HT) and carotid artery stenosis (CAS) were significantly higher in Group 1 than in Group 2 (45 [77.6%] - 120 [62.5%], *P* = 0.04, and 16 [27.6%] - 24 [12.5%], respectively, *P* = 0.01).

**Table 1:** Patients’ demographic, clinical characteristics, and perioperative data.

	Group 1 (n = 58)			Group 2 (n = 192)			
	Min - max or n (%)	Median	IQR	Min - max or n (%)	Median	IQR	*P*-value
Sex, male	43 (74.1)			151 (78.6)			0.58
Age (years)	36 - 82	66	12	37 - 82	59	15	**< 0.01**
Diabetes mellitus	36 (62.1)			105 (54.7)			0.32
Hypertension	45 (77.6)			120 (62.5)			**0.04**
COPD	13 (22.4)			36 (18.8)			0.66
Chronic kidney disease	4 (6.9)			5 (2.6)			0.13
PAD	8 (13.8)			14 (7.3)			0.20
Carotid artery stenosis	16 (27.6)			24 (12.5)			**0.01**
Ejection fraction (%)	25-65	55	20	25 - 65	55	15	0.24
The number of grafts	1 - 5	3	2	1 - 6	3	2	0.62
XCL time (min)	0 - 148	62	43	0 - 197	68.5	43	0.33
CPB time (min)	0 - 254	110	51	0 - 303	110.5	56	0.91
Drainage (ml)	150 - 3000	675	500	50 - 3000	700	450	0.75
Beating heart	8 (13.8)			20 (10.4)			0.63
RBC using	0 - 15	3	4	0 - 14	2	4	**0.004**

COPD=chronic obstructive pulmonary disease; CPB=cardiopulmonary bypass; IQR=interquartile range; PAD=peripheral arterial disease; RBC=red blood cells; XCL=cross-clamping

There was no statistical difference between the groups in terms of EF, number of bypass grafts, cross-clamping (XCL) time, cardiopul-monary bypass (CPB) time, drainage, and beating operation rates. The need for red blood cells (RBC) was higher in Group 1 than in Group 2 (respectively, median 3 - 2, *P* = 0.004).

**Fig. 1:**
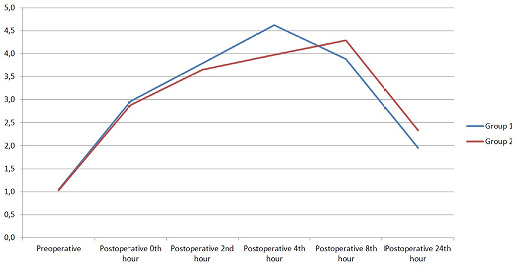
A comparison of serum lactate values between groups. Postop.=postoperative.

A comparison of serum lactate values between groups is shown in Figure 1. Preoperative and postoperative hematocrit and lactate values are shown in Table 2. Preoperative hematocrit values were lower in Group 1 than in Group 2 (median 36 - 38, respectively, *P* = 0.03). No statistically significant difference was found between the preoperative and postoperative lactate values at zero, two, four, eight, and 24 hours between the groups. No statistically significant difference was found between the groups in terms of the difference between postoperative 0^th^, 4^th^, 8^th^, and 24^th^-hour and preoperative serum lactate levels. Similarly, no statistically significant difference was found between postoperative 4^th^, 8^th^, and 24^th^-hour and postoperative 0^th^-hour serum lactate levels. Comparisons between groups in terms of postoperative adverse events are shown in Table 3. Mortality was higher in Group 1 than in Group 2 (6 [10.3%] *vs.* 4 [2.1%], respectively, *P* = 0.01). No difference was found between the groups in terms of the need for reoperation and AKI. CVA was significantly higher in Group 1 than in Group 2 (6 [10.3%] *vs.* 5 [2.6%], respectively, *P* = 0.02). Length of ICU stay and hospital stay were higher in Group 1 than in Group 2 (respectively, median 3 - 2, *P* = 0.00, and median 7 - 6, *P* = 0.00).

**Table 2:** Laboratory parameters.

	Group 1 (n = 58)			Group 2 (n = 192)			
	Min - max	Median	IQR	Min - max	Median	IQR	*P*-value
Preoperative hematocrit (%)	23 - 47	36	8	15 - 50	38	8	**0.03**
Postop. 0^th^-hour hematocrit	23 - 44	30.4	6	16 - 50	31.2	8	0.22
Postop. 2^nd^-hour hematocrit	21 - 43	30.3	6	19 - 48	31.7	7	0.16
Postop. 4^th^-hour hematocrit	23 - 44	29.5	4	22 - 48	31	6	0.07
Postop. 8^th^-hour hematocrit	21 - 39	29.5	5	14 - 43	30.7	5	0.06
Postop. 24^th^-hour hematocrit	22 - 39	28.5	4	16 - 44	28.8	5	0.47
Preoperative lactate (mmol/L)	0 - 2	1	0	0 - 4	1	0	0.72
Postop. 0^th^-hour lactate	1-18	2	2	1-13	2	3	0.47
Postop. 2^nd^-hour lactate	1-15	3	3	1-22	3	3	0.96
Postop. 4^th^-hour lactate	1-15	4	5	0 - 26	4	4	0.77
Postop. 8^th^-hour lactate	1-12	4	4	0 - 27	4	4	0.49
Postop. 24^th^-hour lactate	1- 8	2	1	0 - 29	2	1	0.86
Δ lactate (preoperative 0^th^-hour)	-1 - 17	1	2	-1 - 12	1	2	0.32
Δ lactate (preoperative 4^th^-hour)	0 - 14	3	4.25	-1 - 25	3	3.75	0.80
Δ lactate (preoperative 8^th^-hour)	0 - 11	2	3	-1 - 26	3	3	0.36
Δ lactate (preoperative 24^th^-hour)	-1 - 7	1	1	-2 - 28	1	1	0.71
Δ lactate (4^th^- to 0^th^-hour)	-3 - 7	1	3	-3 - 13	1	2	0.87
Δ lactate (8^th^- to 0^th^-hour)	-6 - 6	1	3	-5 - 14	1	2	0.14
Δ lactate (24^th^- to 0^th^-hour)	-10 - 4-	-5	1	-7 - 16	-1	1.75	0.84

IQR=interquartile range; Postop.=postoperative

**Table 3:** Postoperative data.

	Group 1 (n = 58)			Group 2 (n = 192)			
	Min - max or n (%)	Median	IQR	Min - max or n (%)	Median	IQR	*P*-value
Mortality	6 (10.3)			4 (2.1)			**0.01**
Reoperation	3 (5.2)			6 (3.1)			0.34
CVA	6 (10.3)			5 (2.6)			**0.02**
AKI	5 (8.6)			7 (3.6)			0.11
ICU stay (days)	2 - 36	3	3	0 - 17	2	0	**< 0.01**
Hospital stay (days)	5 - 42	7	4	2 - 30	6	1	< 0.01

AKI=acute kidney injury; CVA=cerebrovascular accident; ICU=intensive care unit; IQR=interquartile range

## DISCUSSION

POAF is one of the most important causes of morbidity and mortality after cardiac surgery. POAF is directly or indirectly associated with many complications after cardiac surgery, such as stroke, bleeding, AKI, respiratory failure, pacemaker requirement, mortality, and prolonged ICU and hospital stay^[1]^. Although there are numerous studies published in the literature on risk factors for POAF, their prevention, or early detection of POAF, its incidence is still considerably high due to multifactorial etiology and unknown reasons. It is the most common complication after cardiac surgery, with an incidence of 15 - 40%^[4]^. Although it is important to return POAF to normal sinus rhythm after it develops, it is more important to identify risk factors and reduce the incidence of POAF before it occurs. Thus, all complications associated with POAF can be reduced. For this purpose, many predictive factors have been investigated so far, but there is not enough study related to lactate levels routinely measured after cardiac surgery. Lactate is a parameter that can be routinely measured in arterial blood gases in the perioperative period, is simple, easily accessible, and has very fast results. Many factors that cause hypoxia at the tissue level can cause its increase. However, there is no definitive information yet about its relationship with POAF. Therefore, in this study, we investigated the relationship of lactate with POAF in cardiac surgery and its predictive importance.

Although there are many publications and recommendations regarding the detection of POAF, no significant decrease in its incidence has been observed in recent years. In our study, patients who underwent isolated CABG were investigated and POAF rates were found to be 23.2%, which is consistent with the literature^[1,4]^. There were no significant differences in baseline demographic characteristics and comorbidities when comparing patients. Only those with POAF were older and had more HT and CAS. Similarly, in the meta-analysis by Mekonen Gdey et al.^[5]^, elderly patients have a higher risk of AF after CABG. XCL and CPB times are among the most important predisposing factors for POAF^[6]^. We did not find any significant relationship between the operation details and POAF between the groups. In the comparison of beating operation rate, EF, XCL time, CPB time, and drainage, no statistical difference was found between the groups. In addition, the number of RBC used in patients with POAF was found to be higher than in those without POAF. We did not find any significant difference between the groups in terms of drainage. However, preoperative hematocrit values were significantly lower in the POAF group than in those without POAF. Postoperative hematocrit values, although not statistically different, were lower in the POAF group at zero, two, four, eight, and 24 hours. In the POAF group, anticoagulant drugs that had to be used in the postoperative period may increase the risk of bleeding and cause the need for more RBCs. In our clinic, blood product use was reduced whenever possible according to Enhanced Recovery After Surgery (or ERAS) recommendations^[7]^. It has been reported that perioperative blood product transfusion may be associated with POAF after CABG^[8]^. In our study, considering that RBC use was higher in the POAF group, this situation may also be related to POAF.

In the comparison of serum lactate values, which was the primary aim of our study, as seen in Figure 1, preoperative lactate values were similar. For postoperative values, lactate levels were higher in the POAF group until the first four hours, but then lower between eight and 24 hours. However, this difference was not statistically significant. In addition, the difference between postoperative lac-tate values and preoperative values was compared. Accordingly, the relationship between the change in lactate values and POAF was examined. However, no significant difference was found between postoperative 0^th^, 2^nd^, 4^th^, 8^th^, and 24^th^-hour and preoper-ative lactate values. Similarly, the lactate difference between other postoperative hour values and postoperative 0^th^-hour value did not differ between the groups.

According to these results, the increase in lactate in the early post-operative period may be related to POAF. However, the decrease in the difference seen in the following hours makes it difficult to make a definitive comment on this issue. It has been reported that POAF is frequently seen within the first week, and the highest incidence is on the 2^nd^ postoperative day, and the highest recurrence is on the 3^rd^ postoperative day^[9]^. Similarly, in our study, POAF was observed in 15 (25.9%) patients on the 2^nd^ postoperative day and in 18 (31%) patients on the 3^rd^ postoperative day. In our study, lactate values measured relatively early in the study period may not be associated with POAF in later days. Although lactate values were higher in the early period in the POAF group, we did not detect a statistically significant difference. Lactate values can increase due to a multifactorial cause. We believe that lactate values alone are not predictive of a polyetiological condition such as POAF. Another result of the study is that mortality and CVA were higher in the POAF group, which is consistent with the literature. It has been reported that the risk of death from all causes is higher in patients with POAF after CABG^[10]^. Similarly, it has been reported that the risk of CVA is higher in patients with POAF after CABG^[11]^. We found no difference in the need for reoperation between the groups. It has been reported that the risk of AF after cardiac surgery is doubled in patients who develop AKI. In our study, AKI was seen at a higher rate in the POAF group, but no statistical difference was found. Finally, the ICU and hospital stays were significantly longer in the POAF group than in those without POAF. Similarly, in the study by Velioglu et al.^[12]^, patients with AF had longer ICU and hospital stays than those without AF. According to these findings, the postoperative results of our study group appear to be consistent with the studies in the literature. All of these results, consistent with the literature, support the negative impact of POAF on postoperative outcomes. Predicting POAF is important in order to reduce its occurrence and associated complications, and to achieve better postoperative outcomes. However, it is clear that more randomized controlled trials are necessary in the future to predict this pathology, which can be triggered by numerous factors.

### Limitations

The most important limitation is that it is a single-center study and has a short follow-up period. The POAF durations and recurrence rates of the patients have not been compared. The most important limitation is that lactate values may increase due to many mechanisms, both hypoxic and non-hypoxic^[13]^. Therefore, we think that further studies are needed for lactate among groups matched with other causes associated with POAF. Finally, considering that POAF is often seen on postoperative days two and three, the lactate values examined in the study often show the period before POAF, not when POAF occurs.

## CONCLUSION

In this prospective study, we found that early postoperative lactate values were higher in patients with POAF than in those without POAF, although this difference did not reach statistical significance. Lactate values alone are insufficient to predict POAF following CABG. However, this issue needs to be investigated in future studies with larger patient numbers and data.

## Data Availability

The authors declare that the database is privately owned by their clinic and therefore cannot be shared publicly.

## Artificial Intelligence Usage

The authors declare that no artificial intelligence tool was used in the preparation of this article.
